# A global view of the aspiring physician-scientist

**DOI:** 10.7554/eLife.79738

**Published:** 2022-09-13

**Authors:** Christopher S Williams, W Kimryn Rathmell, John M Carethers, Diane M Harper, YM Dennis Lo, Peter J Ratcliffe, Mone Zaidi

**Affiliations:** 1 Department of Medicine, Vanderbilt University School of Medicine Nashville United States; 2 Veterans Health Administration Nashville United States; 3 Department of Internal Medicine, University of Michigan School of Medicine Ann Arbor United States; 4 Michigan Institute for Clinical and Health Research, University of Michigan Ann Arbor United States; 5 Li Ka Shing Institute of Health Sciences, The Chinese University of Hong Kong Hong Kong SAR China; 6 The Francis Crick Institute London United Kingdom; 7 Ludwig Institute for Cancer Research, University of Oxford Oxford United Kingdom; 8 Center for Translational Medicine and Pharmacology, and Department of Medicine, Icahn School of Medicine at Mount Sinai New York United States; University at Albany, SUNY United States; University at Albany, SUNY United States

**Keywords:** Scholarly Review Article in Medicine, physician-scientist, training

## Abstract

Physician-scientists have epitomized the blending of deep, rigorous impactful curiosity with broad attention to human health for centuries. While we aspire to prepare all physicians with an appreciation for these skills, those who apply them to push the understanding of the boundaries of human physiology and disease, to advance treatments, and to increase our knowledge base in the arena of human health can fulfill an essential space for our society, economies, and overall well-being. Working arm in arm with basic and translational scientists as well as expert clinicians, as peers in both groups, this career additionally serves as a bridge to facilitate the pace and direction of research that ultimately impacts health. Globally, there are remarkable similarities in challenges in this career path, and in the approaches employed to overcome them. Herein, we review how different countries train physician-scientists and suggest strategies to further bolster this career path.

## The time is now

Arguably no other professional has played a more prominent role than the physician-scientist in accelerating the translation of fundamental scientific discoveries to clinical implementation. This was well illustrated during the COVID-19 (SARS-CoV-2) pandemic, the emergence of which has affected health, commerce, and politics around the globe, and touched virtually every aspect of life in a way not witnessed in recent history. Broad international cooperation accelerated the timeframe from initial recognition of a de novo viral disease to the rapid dissemination of disease characteristics and sharing of research findings in areas such as viral sequences and their drift to different variants, molecular pathogenesis, clinical practice responses, vaccine development, and mitigation strategies; the latter including concepts now familiar worldwide, such as social distancing and masking. Now the task of gaining public confidence and implementing the uptake of vaccines lies ahead. Success has required multinational alliances between clinicians and scientists in government, industry, and academia. Most notably, care of the COVID-19 patient by the physician-scientist has been buttressed by a deep understanding of the underpinnings of disease, acquired through years of dedicated pre-clinical research activity.

These highly skilled workforce members carry dual training, with both their clinical and research skillsets being deployable assets, as observed in the recent pandemic events. In addition to roles in research, because of the urgent need for their clinical expertise at the bedside, many physician-scientist trainees were marshalled into purely clinical roles at the expense of research programs and career development. In the United Kingdom, nearly 90% of such trainees in the Integrated Academic Training Pathway were redeployed to clinical care, impacting research progress, and potentially compromising career aspirations (Wade, 2021; NIHR, 2020). Others were diverted from their original research focus to that of the pandemic to sustain their lab operations and benefit humankind in developing viral testing strategies, vaccines, and antiviral therapeutics. While these and many other such deployments across the globe are well justified, given the dire need for skilled workers, it is notable that globally, the physician-scientist workforce was uniquely impacted by this pandemic (Kliment et al., 2020). Thus, we are at a distinct time to reevaluate systemic processes that attract and support, or disincentivize and deter, individuals from choosing this career path.

Even during nonpandemic times, the physician-scientist workforce is threatened by dwindling funding, heightened clinical and teaching demands, excessive regulation, and the siren call of more lucrative and financially secure work in the private sector. Furthermore, the workforce is aging, the training is inefficient and prolonged, and institutional, industry, private, and government support is, at the very best, inconsistent around the globe (Salata et al., 2018; NIH, 2014). Needless to say, the pandemic is a stark reminder that our younger generation of doctors must be trained both in the art and the science of medicine, in particular, with an eye toward developing talent that can bridge the gap between science and medicine. Toward this goal, we sorely need improved strategies to recruit and support a diverse, equitable, adaptable, and resilient physician-scientist workforce across all career stages. In spite of sobering statistics, this career is one that has intrinsically high value to individuals and to society, and physician-scientists are a segment of the workforce that must be stabilized if we are to rise to combat health threats of the 21st century.

### Value added

Physician-scientists are major research engines that drive discovery across academia, government, and industry. Because of the duality of their career experiences and the blend of clinical and research expertise, they are uniquely situated to recognize gaps in knowledge around clinical care, to gain broad insights into critical aspects of medical physiology based on clinical epidemiology and specific features of disease, and thus, formulate research plans leading to discoveries that would ultimately translate to improved clinical care and overall health (Wade, 2021; Kliment et al., 2020). More than the mere knowledge of dual fields, the stark differences in training prepare these individuals to engage as peers with both scientists and physicians. Beyond bringing recognition to their academic institutions, they are also significant contributors to the biopharmaceutical industry and governmental agencies. Because of their broad perspective and background, they often play educational roles in their home institutions’ clinical and research training activities and are frequently asked to serve in major institutional leadership roles. They are viewed as being positioned to guide the accomplishment of strategic objectives and integration of the tripartite mission of an academic medical center. While the resources, time, interest, or commitment to the physician-scientist pathway may be slowly eroding in academic medical centers, the results of this career pathway continue to provide new basic science insight, medical understanding, behavioral discoveries, and organizational effectiveness. In all, this career provides and supports the evidence-based healthcare system that all nations work to build.

### Broadening scope

Until the latter part of the 20th century, a physician-scientist might have been best described as a ‘practitioner’ who sees patients in a clinical setting and, oftentimes by choice, steps into the laboratory to investigate mechanisms of disease and/or study human physiology. The oldest medical society in the United States—the ‘Practitioners’ Society of New York’—was founded in 1882 on this basis and currently convenes to discuss members’ discoveries. Significant discoveries were the fruits of this model, ranging from a vaccine for smallpox to statins as cholesterol-lowering drugs. Similarly, another United States (US) national society, the Association of American Physicians, founded in 1885 to recognize this unique breed of physician, invites new members in support of creative, collaborative ideation (Carethers, 2019).

**Figure 1.**
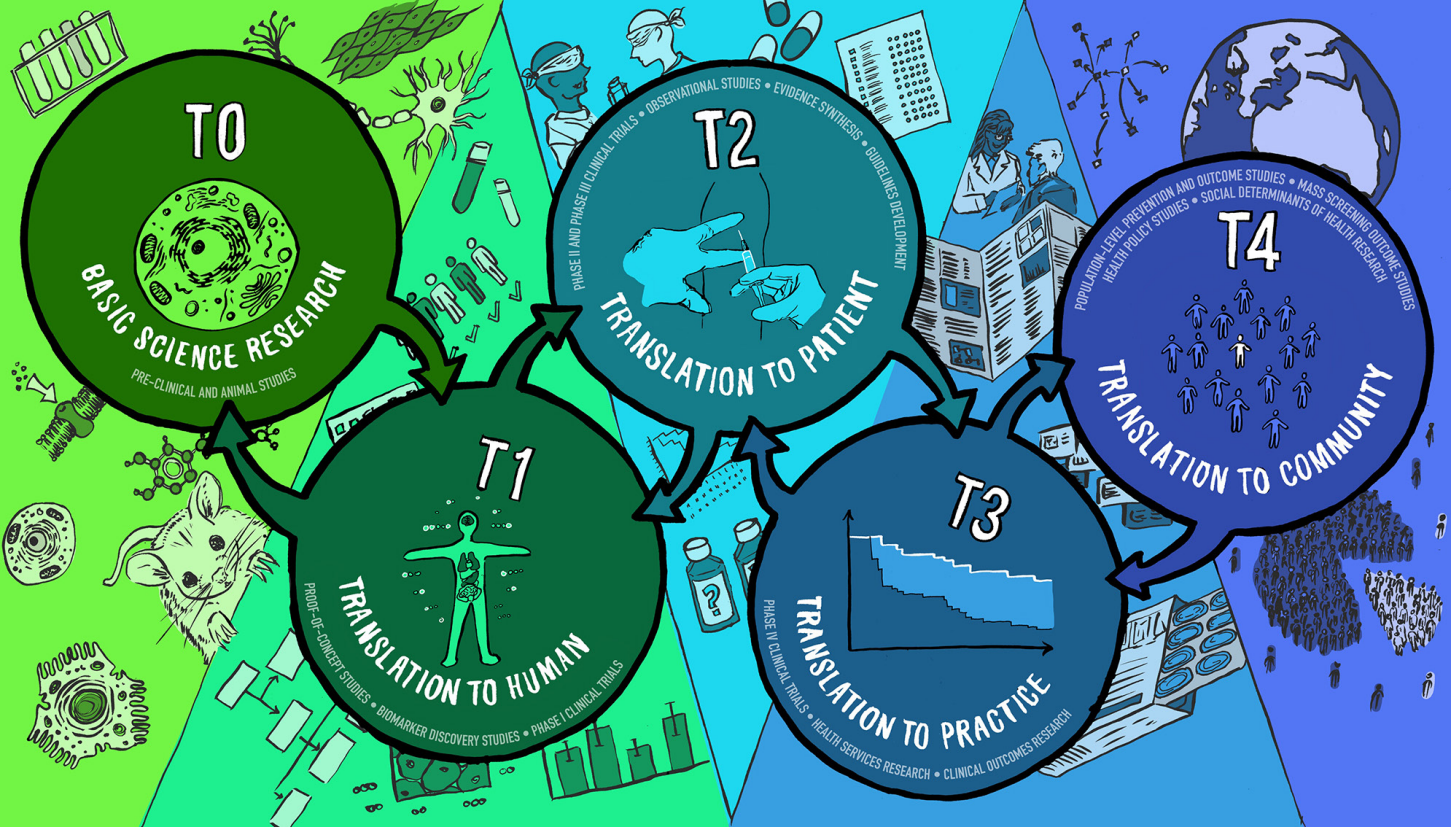
Translational Science Continuum. The T0 pillar anchors basic science bench research, whereas T1 work extends basic science discovery to the first in human trials looking for safety and efficacy endpoints, proof-of-concept, and phase 1 clinic trials. T2 science includes the phase 2 and 3 clinical trials of diagnostics, therapeutics, devices, and other interventions for human health. The physician-scientist must have a different educational focus for this pillar than the T0/T1 physician-scientist. Education must cover clinical trials science, observational studies, meaningful endpoint detection, statistical methods focused on human populations, and human behavior. T3 science extends to phase 4 clinical trials and other observational studies such as health services and clinical outcomes research. Physician-scientists in this arena need education in community-based participatory research and cost-effectiveness and comparative effectiveness research methods. T4 science looks at population-level outcomes and how social determinants of health significantly influence health. Physician-scientists must gain specialization in public policy and health disparities research to include population health guideline development and rigorous meta-analytic strategies.

However, in the 21st century, the scope of a physician-scientist’s research activities has broadened markedly to include not only practitioners who are involved in clinical investigation, but also those focused in areas such as epidemiology, disease modeling, computational medicine, artificial intelligence and machine learning, health services research, population health, and implementation science. The U.S. National Center for Advancing Clinical Sciences (NCATS) was funded in 2011 to increase research productivity that impacts human health (NIH, 2018). Physician-scientists are now involved in all five pillars of translational science (Figure 1). In all, each phase of this translational spectrum has its own distinct impact on human health, and notably each has a different career development path and training requirements to achieve proficiency.

Author and source: Copyright held by Vivid Biology. The figure is available under the terms of the CC-BY Attribution 4.0 license: https://creativecommons.org/licenses/by/4.0/.

All clinical specialties have, and should continue to benefit from, rigorous scientific inquiry led by well-trained physician-scientists, thus improving overall patient health through discovery. Clinical training opportunities in specific disciplines are mandated by the appropriate licensing board and divisional and departmental leadership. Trainees should be able to readily identify those programs that are resourced, structured, committed, and with faculty and mentors to maximize the chances of the trainee successfully launching and maturing their own research programs. To this end, in the context of the US, the Association of American Medical Colleges (AAMC) Training Opportunities for Physician-Scientist (TOPS) Committee was created in 2019 to ‘Provide information and resources to trainees and MD/PhD Directors on postgraduate physician-scientist training activities, programs, and resources.’ The TOPS Committee developed a physician-scientist training program (PSTP) webspace containing discipline-specific content, informational webinars, and program listings providing valuable information to trainees and program directors. Our trainees should be able to use this information to identify those programs that best match their training needs and expectations and maximize their chances for continued success in this career path. Additionally, several US programs at the divisional and departmental levels have obtained competitive National Institutes of Health (NIH) T32 Training Grants that allow small groups of aspiring postdoctoral specialists to have research-protected time to develop investigative skills during their Accreditation Council for Graduate Medical Education (ACGME)-governed training. The overall goal of this NIH-funded training is to move to a Career Development Award (CDA), and subsequent independent competitive grant funding as the physician-scientist evolves.

It is also important to recognize that in specialties that require rigorous ‘craft’ training, maintaining flexibility, and creativity in developing training opportunities is paramount. For example, in some specialties, it may be more efficient to fully complete clinical training prior to embarking in research activities. At the Crick Institute in London, of note is an increase in research career interest in young, fully qualified surgeons, and the Institute has therefore developed programming to support these highly specialized ‘late bloomers’. Another recent opportunity developed in the United Kingdom is the Medical Research Council’s ‘clinical academic research partnership scheme’ which supports fully trained NHS consultants in research activity.

## The global physician-scientist pathway

### Student training

Mechanisms for training physician-scientists vary widely from country to country. In the US, combined MD/PhD programs have been in existence since the mid-1950s, with formal Medical Scientist Training Programs (MSTP) beginning in 1964. In 2021, these dual degree programs matriculated 750 students, had an enrollment totaling 5,913 trainees in 98 medical schools, and graduated over 660 trainees (AAMC, 2021). Roughly half of the programs are supported by the NIH through a T32 mechanism from the National Institute of General Medical Sciences (NIGMS); this provides financial support and consistency in training activities across the nation. These programs integrate clinical and graduate training and interweave additional career development activities over the training course. Trainees typically spend two initial years in biomedical science training, enter graduate school for doctoral studies, secure their PhD, and thereafter return for two final years of clinical training. A few programs, notably Duke University School of Medicine and Vanderbilt University School of Medicine, have 3-year clinical curricula, thus reducing time to degree. While these programs have created a widely recognized pathway for training, the success of combined degree programs can deter medical students and MD trainees from pursuing research opportunities. Over the past several years, many medical schools are evaluating and revamping clinical training curricula, with a goal to create additional opportunities for innovation and further avenues of physician-scientist training. However, it is important to note that faced with an increasingly complex medical curriculum, many schools have transitioned to flipped-classroom, case-based, and professional-skill-focused course design, raising among other concerns, the deficit of core biomedical science training thus rendering traditional MD students ill-equipped to pivot into a research direction.

The levels of formalized support for training physician-scientists also vary from country to country. The Canadian physician-scientist training model, previously funded by the Canadian Institutes of Health Research (CHIH), closely approximates the US MSTP (Twa et al., 2015; Lewinson et al., 2015; Strong et al., 2018). In 2015, the CHIH eliminated this program, leaving an entire generation of trainees with unclear training paths while individual institutions cobbled together support to attempt to maintain programs, in essence, profoundly jeopardizing the physician-scientist pipeline in Canada (Twa et al., 2015). Australia lacks a nationally sponsored physician-scientist training strategy (Eley and Benham, 2016); however, a review of student perspectives revealed an intense interest in pursuing research among Australian medical students (Eley et al., 2017). A survey of trainees indicated that perceived barriers included the absence of a clear, consistent pathway, formal funding mechanisms, time away from the clinic, and purposeful mentorship (Eley et al., 2017). Despite the lack of a formal physician-scientist track, most medical schools facilitate combined MD/PhD training in an intercalated curriculum (Eley, 2018), similar to the US structure. However, the time spent in the graduate training phase is completed in 2–3 years, bringing the total training time to between 6 and 7 years—which is 1–2 years shorter than the US training equivalent. Typically, trainees in these programs are one-offs, with most not having formal pathways or structured training (Eley et al., 2017). Germany and France offer integrated MD/PhD training often between the second and fourth years of primary medical training; in France, there is also an option to pursue a master’s degree during medical school and PhD training during residency (Noble et al., 2020). In Germany, compensation is provided during this time; however, at a reduced rate in comparison to postdoctoral fellow salaries. Nonetheless, in the German system in general, there is limited medical school debt (Bossé et al., 2011), which is in stark contrast to 2021 U.S. Medical School graduates, who accumulate an average of $215,900 in Medical School debt (Hanson, 2021).

In the United Kingdom (UK), a small number of medical schools offer MD/PhD programs and a larger number offer an intercalated year of scientific training often involving ‘hands-on’ research as part of a BSc degree. Though these opportunities are well subscribed, they come with the cost of increased fees to the trainee, which may discourage or exclude many able and gifted individuals. Doctoral (PhD) training in the UK is more commonly undertaken after medical graduation and there is a semistructured route by which the NHS, through the National Institute for Health Research (NIHR), provides both pre- and post-PhD support for protected research time, which is integrated with clinical training. Most commonly, PhD studies are supported by a specific ‘Research Training Fellowship’ funded by the Medical Research Council or one of the UK’s major medical charities, including the Wellcome Trust. Despite these opportunities there remains a concerning attrition rate with relatively few of those successfully completing their PhD being able to sustain a long-term career as a physician-scientist. Pressures of maintaining high-intensity NHS clinical practice and, in some cases, excessive regulation based on assessing levels of clinical exposure, as opposed to competence, are problematic. There is also a perception that the funding of independent early career investigator positions has become overly cautious and overly dependent on research ‘track record’, which is more difficult to achieve in parallel with clinical training. Recognizing this restraint, some of the UK’s major research institute’s such as the Francis Crick Institute are now offering such positions on a fixed term basis. For instance, the Francis Crick Institute offers 6 + 6 (6 years renewable for 6 years) appointments with the intention that these individuals will then revert to University Hospital Medical Centers to populate the leadership of the physician-scientist community.

In Hong Kong, two medical schools have developed curricula that allow a proportion of their medical students to acquire additional research training. For example, at the Chinese University of Hong Kong, there is an option for 20% of the medical students, with the highest admissions scores, to pursue a Global Physician-Leadership Stream in which a student is paired up with an academic mentor and is attached to a laboratory or assigned a public health project throughout the duration of the medical curriculum. For the University of Hong Kong, year 3 of the 6-year medical curriculum is referred to as an ‘Enrichment Year’. During this year, students can pursue service/humanitarian work, research attachment, and intercalation. A proportion of such students can choose to pursue a Master of Research in Medicine through additional coursework, research training, and submission of a dissertation. For medical students who wish to pursue a PhD degree during their studies, the Croucher Foundation, a charitable foundation devoted to funding scientists at different stages of career development, represents a potential source of funding.

### Clinical training

Several specialty boards in the US recognize the need for specialized physician-scientist training structures. Examples of this include the American Board of Internal Medicine (ABIM) Research Pathway, the Holman Research Pathway in Radiation Oncology (ABR), and several American Board of Pediatrics (ABP) variations, to name a few. Recognition that maximized gains are realized by coupling these pathways with departmental and institutional programs has led to the creation of PSTPs (below). These provide community, mentoring, funding, and leadership for aspiring physician-investigators. Additional formalized programs for entry into a physician-scientist training path are emerging as Research in Residency (RiR) programs, and training programs that equip later stage housestaff and fellows with the time and resources to train in a scientific discipline as postdoctoral fellows. The funding strategies and guidelines for these approaches are disparate, ranging from NIH-funded programs under the R38 ‘Research in Residency’, supported by four NIH institutes and the more broadly supported T32, and K12 mechanisms (targeting residents, fellows, and junior faculty, respectively), as well as foundations or institutional resources that have filled the gaps. For example, the Burroughs Wellcome Fund offers the highly flexible Physician-Scientist Institutional Award, aiming to recruit MD-only clinicians into basic science careers, as well as the individual Career Awards in the Medical Sciences (CAMS), providing funding during the critical fellow-to-faculty transition years again for MD-trained investigators. The Robert Wood Johnson Clinical Scholars Program offers 2 years of fellowship training geared toward competencies needed for the T2–4 scientific work (Figure 1). Other NCATS-sponsored training programs are the postdoctoral TL1 programs and the early career KL2 programs that are institutionally based. In addition, in the US, there are varied possibilities for postdoctoral training of mature physicians after completion of their clinical training. Prominent among these is NIH’s own Intramural Research Training Program, as well as multiple extramural support structures, including foundation funding. One example is the Damon Runyon Clinical Investigator Award, a program that is exclusive to MD-only would be physician-scientists. Such foundation-derived support is critical to identify and foster talented individuals who are later to recognize the career direction.

To combat the decline in the physician-scientist workforce, Germany recognized the need for structured, consistently funded, institutionally based programs during residency training. In 2015, the German Research Foundation (DFG) announced the Clinician-Scientist Program to provide medical residents with 3 years of protected time to engage in research after their second year of residency. This uniquely structured program is designed to run concurrently with residency training and balances 40–50% protected time, each for research and clinical training, which includes a curriculum of research training modules.

Hong Kong has a longstanding shortage of doctors, with approximately 2 doctors *per* 1,000 population. This level is below that in Singapore (2.5), the UK (2.6), and the US (3.0). As a result, it has been a challenge to create protected time for junior medical doctors to pursue research training. Two medical schools in Hong Kong have employed selected trainees under the titles of Clinical Lecturers or Clinical Practitioners to allow them to develop their academic interests, while acquiring the necessary clinical experience toward qualifying as a specialist. Specialist training in Hong Kong is governed by 14 colleges for the various medical specialties and one dental college, all under the auspices of the Hong Kong Academy of Medicine. Most clinical academics obtain their Clinical Assistant Professorships after completing their specialist qualifications. Like the US, foundations provide an important stopgap to support individuals at the earliest stage of the physician-scientist career, one example being the Croucher Foundation which also funds a number of clinical assistant professorships.

### Common threads

From the above review of physician-scientist training across a select number of nations, common themes that pose threats to systems emerge; these need to be accounted for in driving changes that invite and support more talented individuals to pursue this career path. Finding the time to train dually in high-level disciplines and securing funding are at the crux of the issue. Perceptions that a physician-scientist career is unsustainable or that it must be prescribed in the form of MD/PhD training prevent talented individuals from even considering the path. Academic physician-scientist retention is distressingly low regardless of the mechanism of training, which further strains a system that is resource-limited already. The additional drain of talent to industry jobs is a further challenge. While this is a valuable and viable career path, the cumulative effect is to reduce cohort sizes in training centers, resulting in a deficit of visible role models and mentors thus feeding a vicious cycle of attrition.

Core features critical to success are also readily discernable and present in nearly every country’s program. Intense, immersive, dedicated longitudinal training in both the clinical and scientific arenas is foundational. This must be led by dedicated faculty with domain-specific expertise, commitment to training, and passion for mentoring. Funding is derived primarily from institutional or governmental sources, and while oftentimes the institution is forced to absorb a significant component of the overall training costs, there are emerging programs directed to this hurdle. The coordination of support mechanisms is an important strategy for the future. Efforts to ensure that cohorts of trainees form a community that fosters near-peer and peer-peer mentoring are central to virtually all programs. Presentations at national and international meetings, publication in peer-reviewed scientific journals, and advancement from programs dedicated to training physician-scientists to independent research identified by garnering competitive funding represent the primary metrics by which trainees’ long-term potential is adjudicated worldwide.

### A risk for derailment

In the US, predoctoral training of physician-scientists *vis-a-vie* NIH-supported MSTPs and other MD/PhD programs have traditionally had a high graduation success rate. Indeed, with improvements in the training process, the attrition rate has steadily reduced over the past several decades. These improvements leverage existing, established curricula for training clinicians and scientists, with decades of experience in accomplishing both objectives. Indeed, contributors to the reduced attrition likely are the value-added, supra curricula components of these programs—notably, building communities, fostering near-peer and peer-peer mentoring, normalizing the experience of dual training, administrative support, and dedicated faculty with formal designated ‘effort’ who are charged with shepherding class after class through the rigorous predoctoral training process and its inherent challenges. Trainees are immersed in an integrated curriculum, where the excitement of discovery is coupled with clinical coursework and experiential training via time on the wards.

### Immersive, intense, clinical training

In the US, upon graduation, the vast majority of MD/PhD trainees advance to residencies. Here, they are immersed in intense clinical training with relatively little time for research and with a curriculum that is predominantly protocol/guideline based, and largely defined by national medical board standards. While faculty in these programs no doubt provide outstanding tutelage in clinical care, they may be less well versed in the challenges a new physician-scientist graduate may face at this critical career stage. In this regard, the PSTP programs, as an adjunct to the formal graduate medical experience, provide a way forward into the research arena after or during the completion of graduate clinical training, with on-ramps for both MD and MD/PhD graduates.

The increasing complexity of clinical care, coupled with the near-universal expansion of administrative requirements affecting patient care, overseen by rigid, time-based board-dictated training requirements, leaves little time for reflection, intellectual pursuits, or the nurturing of curiosity—the very underpinnings of scientific discovery. Yet, there is undoubtedly, for many, immense and significant proximate gratification in caring for patients. In contrast, the rewards for engaging in science are very different and are often less tangible. One can put untold hours into a research project, only to have the cherished hypothesis test false, or even worse, prove to be untestable. Success in clinical diagnosis and patient care and the appreciation of patients at this stage can contrast starkly with negative manuscript reviews and grant reviews that are more the norm for scientific peer review, as well as the uncertainties of future stability inherent in academic-research-focused careers. Physician-scientist trainees are particularly vulnerable at this career stage making mentorship, coaching, and sponsorship essential requisites to their support.

### Life happens

This career stage also coincides with increasingly complex personal life activities. Depending on the time spent in the laboratory—which can vary between 3 and 6 years—some MD/PhD trainees are in their mid-thirties by the conclusion of residency training. Many may have started families or have stewardship responsibilities with aged or ailing parents. This oftentimes results in a reflective, but thorough evaluation of their career goals, with renewed recognition of differences, perceived and otherwise, in financial security between purely clinical and research-based careers, or even worse, the realization that there is modest financial security in research careers, in comparison to lucrative, stable clinical careers, *albeit* demanding. In certain large non-university medical centers with expansive clinical outlays—which focus predominantly on the clinical mission as a driver (and determinant) of research activities—it is relatively rare to have MD/PhD-trained physician-scientists on faculty in clinical departments, further limiting the role models for this career. It is not uncommon for physician-scientists trying to perform solely in a patient-centered research domain to drift into pure patient care, which in many settings is the primary means to fund salaries. Such goal misalignment can lead to attrition, frustration, and burnout.

In contrast, certain hospitals and medical centers in the US have traditionally been ‘incubators’ for aspiring physician-scientists who are supported by stable salaries and opportunities to create their own research programs. One example is the Veteran’s Affairs (VA) hospital network, which provides a unique version of this stable setting in which to launch an independent research career through CDAs that provide substantial support and prepare early career investigators for additional funding mechanisms, such as the VA Merit Awards that sustain independent careers (VA Career Development Program, 2015).

### Compensation challenges

The issue of compensation is a challenge that commonly affects physician-scientist careers across the globe. Holding the same credentials as their clinical colleagues, physician-scientists typically make far less than a well-compensated busy clinician. In some cases, the disparity can vary by orders of magnitude. On the other hand, MD/PhD physician-scientists also hold the same credentials as their PhD scientist colleagues; however, in this case they typically take home salaries considerably higher. Where this middle ground is ‘right’ is a challenge for administrations because physician-scientists often bring a special value to teams in in spite of the smaller clinical revenue they generate. They also bring a clinical gravitas to basic and translational research. The real challenge comes from the sources of compensation.

In the UK until recently (the last 10 years or so) academic physicians were compensated reasonably well under a ‘merit award’ scheme that involved competitive application to a national body. This however is now much more focused on NHS performance so that there is now an emerging problem for those whose main nonclinical contribution is research. Universities try to compensate to make up the gap; however, there is often no pay scale and arrangements are ad hoc. In Japan, compensation is very directly pegged to clinical activity; thus, research time occurs after clinical duties are completed and often constrained to nights and weekends (Imai, 2021).

For NIH-funded investigators, the NIH salary cap of $203,700 in 2022 represents a salary that is not sufficient to even cover the career spectrum of PhD scientists. Physician-scientists in the US thus live in two worlds: calculating the percentage of effort devoted to various NIH-funded duties within a scale of 0–$203,700, while earning a salary that can vary wildly above the NIH set scale. Several organizations, such as the MGMA and AAMC, tabulate physician salaries, but the data are not easily translatable for physician-scientists. When a physician earns a salary that is twice as high as the NIH cap, where does the extra money (plus fringe benefits) come from? It easily becomes obvious why some types of physicians particularly are discouraged from pursuing this career path. It is even easier to see why members of groups underrepresented in medicine would be dissuaded from even considering such a career decision that seriously compromises their earning potential (Kalet et al., 2022). An unseen group, is the late bloomer, a physician who develops a talent for research later in their career—are we forced to reduce their salary, or expect them to continue to maintain their clinical productivity while building a research program? Forces of incentive have created the discrepancy we see today, with a greater proportion of physician-scientists in fields where the compensation differential is less significant; physician-scientists are rarely encountered in those fields where the differential is large. Moreover, our models of training have created a workforce that is now largely limited to those who accepted the state of affairs early or were financially able to accept that future.

### Current interventions

American specialty boards recognize that a modified clinical training path best supports physician-scientist careers. In 1985, the American Board of Internal Medicine (ABIM) allowed integrated research and clinical training during residency in select institutions. Formalized as the ABIM Research Pathway in 1995, this track shortens residency training by 1 year and provides up to 3 years of research time during fellowship training, similarly to certain European tracks (above) (Todd et al., 2013). The select track offers protected time for trainees to engage in meaningful scientific pursuits and lays the foundation for an independent research program for the trainee. Program outcomes have been excellent, with 91% of pathway graduates reporting continued research engagement with 85% having obtained competitive independent extramural funding (Todd et al., 2013). The American Board of Family Medicine is initiating a similar research track for residents interested in T2–T4 translational research (Doubeni et al., 2017).

Given the considerable variability in clinical training requirements between specialties, it is not surprising that many PSTPs began as departmental entities with significant variability in program design that take advantage of institutional strengths. Some longstanding programs in the US include the Specialty Training and Advanced Research (STAR) Program at the University of California, Los Angeles (UCLA) (which also offers a PhD) (Wong et al., 2016), Washington University’s Oliver Langenberg PSTP, and the Vanderbilt University Medical Center’s Harrison Society. These programs typically span residency and fellowship, encouraging trainees to develop longitudinal relationships at a single institution, connect budding physician-scientists, and foster community formation to facilitate near-peer and peer-peer mentoring.

Recognizing that critical components of physician-scientist training are discipline agnostic and that key training elements could be centralized and thus not only increase efficiency, but also build larger communities and foster institutional alliances, several institutions, such as Johns Hopkins, Northwestern, UCLA, and the recently launched Duke and Yale programs, have invested in institution-wide physician-scientist training initiatives that span all clinical departments (Permar et al., 2020). Such programs are often referred to as ‘umbrella’ programs and have common core programming, such as seminars, retreats, and research forums, and even provide research funding. This specialty ‘agnostic’ structure allows intermixing of physician-scientist trainees from multiple career stages in different clinical specialties, thus fostering cross-fertilization and sharing of best practices between trainees and faculty. It also enhances the increasingly transdisciplinary nature of scientific investigation focused at improving human health.

Scholarly activity is a required component of most residency training programs. A comprehensive nationwide survey of US training programs identified wide departmental variability in the frequency and the nature of training opportunities; however, formal, structured research tracks have led to greater trainee research engagement (Ercan-Fang et al., 2017). Standardized postgraduate physician-scientist curricula, allowing for the scalable implementation of structured programs tailored to institutional strengths and weaknesses, should improve the quality of these experiences, and allow us to identify and cultivate individuals with newly found research aspirations.

## Potential interventions

### 1. Teaching the “Hidden” Curriculum

The American Society of Clinical Investigation’s (ASCI) mission is to ‘support the scientific efforts, educational needs, and clinical aspirations of physician-scientists to improve the health of all people’. In 2019, ASCI charged a working group consisting of MD/PhD and PSTP Directors and physician-scientists from different clinical specialties to identify novel ways of further supporting physician-scientist careers. Integrating the working group’s output with data from a recent survey of highly accomplished early-career physician-scientists recognized by the ASCI as Young Physician-Scientist Awardees determined that physician-scientist trainees, at all stages, will benefit from formal training in what was termed as a ‘Hidden Curriculum’ (Table 1). While programs at some institutions provide training in select areas, standardized, structured programming activities, common to all programs, would offer significant trainee benefits. The Research Committee of the Alliance of Academic Internal Medicine, after a rigorous assessment of opportunities and challenges, published six key essentials that constitute ‘Best Practices’—one of which was mentoring (Blanchard et al., 2018; Williams et al., 2018). Increasingly, intentional training in these elements of the ‘Hidden Curriculum’ is becoming essential in training program plans.

**Table 1.** The Hidden Curriculum.

1	Networking skills
2	Mentor training
3	Research Management
4	Promoting Science
5	Resiliency
6	Diversity, Equity, and Inclusion
7	Team Science

### 2. Derisking innovation

One challenge facing the field is how to prepare emerging investigators for a career that is inherently risk taking, when many may have chosen medicine as a path with job security, and which is taught with an eye toward choosing the path of least risk and greatest benefit to the patient. Physician-scientists need opportunities to practice and regain comfort with taking scientific risk even if it increases potential failure. Our training programs and mentors need to develop skills that align with strategy and research with comfort levels in assessing and taking risk.

Risk is also a structural barrier to the expansion of the physician-scientist pool. First, regarding the individual, institutions, training programs, and mentors must be prepared to invest in a bright individual with a good question before a clear ‘track record’ has been established. Second, the infrastructure and the individual investigator must be prepared to invest in what is inevitably a new area of research, one which may not be currently fashionable. Our culture should support and challenge clinicians to ask new questions based on their somewhat difference perspective on biomedical sciences. Training programs need to teach risk assessment and foster environments that can embrace hypothesis testing that sometimes proves the null. It is critical, particularly in an era where funding thresholds fluctuate, that study sections and grant reviewing bodies recalibrate and recognize the value of risk taking, especially from proposals from early-stage individuals. This has been recognized by more liberal NIH funding thresholds for new investigators and a plethora of foundational supports that recognize the need to engage these early career researchers. Importantly, those medical schools that prominently display their interest in promoting research must actually do so by taking the extra step to support high-risk, high-reward research from individuals at all career stages, particularly for the cadre of young physician-scientists.

### 3. Accelerating time to independence

Over the past 50 years, the MD/PhD training time to degree has steadily increased, at least in the US, going from 6.2 years before 1975–8.0 years in the cohort trained from 2005 to 2014 (Brass and Akabas, 2019); it currently stands at 8.2 years. The time to first R01 has also increased substantially (Lauer, 2021). This is despite the implementation of programs to shorten or increase overall training efficiency, such as licensing board sanctioned research pathways. The progressive lengthening of the training dwell time of this workforce continues to have a significant impact on recruitment and retention. As noted above, trainees are in tenuous career positions at later ages, with increasing responsibilities and financial demands, with lucrative, more secure options readily available in clinical practice or alternative career paths. To counter this obvious problem, strategies to optimize predoctoral training need to be investigated. Traditional US MD/PhD programs are modeled in the 2-4-2 fashion, wherein two preclinical years are followed by a variable graduate training phase bookended with an additional 2 years of clinical training. Duke and Vanderbilt University Medical Schools have an adjusted curriculum in which the first 2 years are optimized to a single preclinical year, followed by the core clerkships (typical third year of medical school in most programs), followed by only a single final year of medical school, thus shortening time to degree to 7.3 years on average (Vanderbilt)—importantly, with excellent match results into research-based residencies. Consideration for modifying and adopting similar curricula nationally will reduce time to independence.

Faced with mandates to ensure rigor in PhD training, many PhD components of MSTP programs, require scholarly output, usually defined as a primary author manuscript. With the ever-increasing requirements for publication, the bar for publications, and for publication in high-impact journals, has risen precipitously. A process that was originally designed to teach structured problem-solving, instead can become focused on achieving a particular product in the form of a publication. We recently surveyed residency program directors and confirmed that a primary authorship is perhaps the most impactful aspect of training (Gallagher et al., 2022). However, the uncertainty and unpredictable nature of the process of publication can present a misalignment of goals. With few clear metrics by which to adjudicate a trainee’s progress, it is not surprising that graduate school dwell time is vaguely interpreted. In response, several graduate programs are adapting lessons learned from undergraduate medical education (UME), exploring the use of competency attainment, measured by milestone assessments, to determine whether the student has acquired the core competencies critical to scientific training and career success during their PhD training (Verderame et al., 2018). Similar to efforts graduate (PhD) competencies, a recent report suggests a framework for physician-scientist competencies relevant for MD/PhD training programs (Estrada et al., 2022). Competency-based graduate medical education (GME) could shorten time to degree for select individuals and provide some much-needed guidance to faculty and trainees alike.

Similarly, historical and current medical licensing paradigms are also time based. However, knowledge acquisition is only partially measured and/or dependent on how long a trainee has participated in residency. Given the progressive increase in time to independence for physician-scientists, reductions in total training dwell time could be achieved by aligning licensing with UME and GME competency-based criteria for achieving board certification (Verderame et al., 2018; McClarty and Gaertner, 2015). This would mean that the award of medical licensure is based on attaining competency, as opposed to training stage dwell time.

### 4. Innovation in funding

Successful physician-scientist careers require longitudinal, durable support across their training spectrum. Governmental agencies, such as the NIH in the US and MRC in the UK, private foundations, and academic medical centers have shouldered most of the costs of training physician-scientists. This workforce is highly sought after by the biotechnology and pharmaceutical industry, who recruit top-tier talent that are products of PSTPs. The time has come to embrace physician-scientist roles in a multitude of positions, and pipelines to the pharmaceutical industry may benefit from focused programs. Partnerships forged with industry may increase trainees’ access to role models in industry, accelerating preparation for a career in the public sector, and expanding the trainee pool rather than cannibalizing a scarce resource. New models of training that incorporate elements of business and management skill training might be envisioned to prepare physician-scientists for unique elements of the pharmaceutical industry. Industry involvement should consider committing to establishing transparent, and importantly, unencumbered lines of funding toward the growth and development of the physician-scientist workforce that is prepared to meet the needs of academia, industry, biotech, and government opportunities of the future.

### 5. Changes in publishing trends

Historically, translational science did not attract interest from top journals and was perceived to be undervalued in the promotion process. Given the prominent role of publishing and career success—‘publish or perish’—it is not surprising that most physician-scientists have been drawn to developing basic science research programs, for some years now. The evidence that supports national health policies appears in journals that disseminate research outcomes from many perspectives. Physician-scientists need journal support to publish all of the pillars of translational research (Figure 1). Publishing houses, such as Springer and Elsevier, have created journals that focus specifically on the multiple translational pillars. Perhaps, this trend in publishing may yield physician-scientists having a more translational or clinical slant to their careers. On the other hand, clinical research has also become more broadly mechanistic and hypothesis driven, with sophisticated translational studies accompanying clinical investigations. This shift should allow closer alignment and narrow the gap between clinical and research activities.

Another major trend relates to democratization and dissemination of scientific and clinical investigation—openly, rapidly, and in a manner where journal articles become the currency of trust in medicine. The concept of ‘publish, then review’ (Eisen et al., 2020) which journals such as *eLife* are leading, represent a momentous change in which authors will have control over when their work is published as a preprint on Bioxriv or Medxriv, and later be reviewed and curated. This new process will divorce the dissemination of reviewed science, which will take form of a ‘Refereed Preprint’ and its publication. While debated in some circles, this culture change is particularly useful for the physician-scientist, whose career trajectories are limited by time to publication.

### 6. Fixing the compensation gap

How do we rectify the appropriate compensation model, and the source of funding to support salaries that recognize the contribution of clinical researchers? The problem is twofold. The first is that identifying the appropriate salary for such work requires real economic analysis, factoring in the value of incentives to draw talent into the field, rather than depending on the reward of discovery being enough. The second problem is finding solutions for those with hiring and salary-making authority, who are faced with limited budgets and often high targets for productivity. This is a complicated problem of value, reward, and incentive, that will require high-level discussions. One possible solution includes reassessing the NIH salary cap to recognize the scale for physicians engaging in research. Considering alternative methods for assigning value and pay, the VA, for example, has adopted a pay scale and assigned effort for work that more effectively harmonizes the work of full-type physicians and physician-scientists. The US Department of Defense provides considerable funding for research, and assigns budgeted effort according to actual pay, such that grantees sort out the issue of effort and grant fund distributions themselves. Finding alternative sources to offset portions of salary not covered by clinical effort is also a strategy applied to large philanthropic gifts, or can be provided by foundations, although this mechanism can be unequitable. Beginning by creating national guidelines, such as exists for practicing physicians, would also help level the playing field, and give physician-scientists leverage in negotiations or setting expectations. This is an area in need of serious attention; our future workforce needs talent from those who cannot afford to make tough financial choices, from those who had not anticipated the career early enough, and those who have expertise in the widest range of medical specialties.

### 7. Promoting a diverse worldwide physician-scientist workforce

An increasingly diversified population should result in increased representation in the physician-scientist workforce; however, structural, systemic, and cultural barriers exist that limit entry or reduce retention in this career path (Behera et al., 2019). Women, the economically disadvantaged, individuals with disabilities, and underrepresented trainees are especially vulnerable to attrition from the physician-scientist path unless there is equity recognition. Disruptions in research activities, collaborations, publishing, and administrative staffing have affected many scientists during the COVID-19 pandemic, with women, early-stage investigators, and those from diverse backgrounds being disproportionately impacted (Kliment et al., 2020; Johnson et al., 2021). The burning question therefore is—how can diversity be improved (Table 2)?

**Table 2.** Strategies to Promote Diversity.

1	Institutional anti-racism policies
2	Support URiM trainees and faculty
3	Promote diversity in public for a and institutional leadership
4	Provide child/elder care subsidies
5	Track diversity outcomes metrics
6	Develop ‘diversity aware’ training curricula

First, the community of leaders must apply a zero-tolerance anti-racism policy, with clear guidance on how exactly to promote diversity, equity, and inclusion. Many medical schools are applying these principles in earnest and recognize that more needs to be addressed, while others fail to practice their own principles. Second, academic institutions should purposefully identify, retain, and promote not only underrepresented minority trainees, but also faculty members, so that trainees can readily identify role models. Third, there should be a concerted effort to diversify representation in speaker lineups and panel representation at local, national, and international conferences, as well as diverse representation in an institution’s leadership so that trainees see people ‘like them’ succeeding in academia. Fourth, there needs to be agreement that diversity yields rich rewards and highly values national healthcare objectives. Fifth, academic medical centers should provide support mechanisms for young families during training with services, such as subsidized child- or elder-care support. Sixth, and importantly, diversity should be tracked as outcome metrics from training programs. And finally, formalized training in identifying ‘differences in training needs’ for individuals from diverse backgrounds should be included in the training curricula (i.e., Culturally Aware Mentoring) for faculty, staff, and importantly, trainees—this will help the next generation learn from these lessons early in their careers (Carethers, 2019; Carethers, 2020).

### 8. Simplifying internationalism of the physician-scientist global workforce

There is an acute need for internationalism in the acceptance of professional qualifications. There has been considerable adverse movement in this over the last decade or so—namely that it is now much more difficult for a physician-scientist (who will likely be maintaining <50% clinical practice) to gain a license to practice in another country due to increasingly stringent local licensing requirements and contracts with reimbursing agencies. It could be argued that this is a time of crisis for internationalism and that it would be eminently feasible (and to the advantage of all parties) to allow international recognition of a license to practice for individuals with internationally recognized skills and qualities—this could be adjudicated by the relevant national bodies for example, Academy of Medical Sciences in the UK. Unfortunately, licensing in the US remains state specific, so that in some states it may be difficult to rationalize international recognition. In New York state for example, passing the membership examination of the Royal Colleges of the United Kingdom and Ireland are regarded equivalent to 3 years of postgraduate training in the US. Acceptance of professional qualifications at the international level lowers another barrier to vitally important exchanging of talent between countries and in response to crisis and needs.

### 9. Conceptualizing an international physician-scientist training society

Professional societies provide a forum for colleagues to interact, share ideas, make connections from seemingly disparate events, and importantly, foster and inspire careers of trainees and more junior colleagues across all the translational research spectrum. Professional societies can provide specialized support at grander levels, meeting the unmet needs of their constituents. For example, the American Society of Clinical Investigation (ASCI) recognized that specialized support might bolster the careers of PSTP trainees and early junior faculty recently launched the Emerging Generation Award (E-Gen) in 2022 to provide recognition, support, and encourage attendance at the national meeting with programming structured to foster the career development of physician-scientist trainees at these early career stages. The Association of Physicians of Great Britain and Ireland (AOP), founded by Sir William Osler in 1907, among other objectives, seeks to ‘develop the careers of translational researchers’ (West, 2007) and provides grants for junior investigators to enhance their career development. In 2016, the Interurban Clinical Club in the United States, also an Oslerian society formed in 1905, for the first time in its history allowed MD/PhD students to participate and network during what had otherwise been a formal event that showcased the scientific achievements of the most senior investigators (Forrest, 2016). Likewise, the formation of an international physician-scientist society would allow sharing of knowledge and best practices; facilitate near-peer and peer-peer engagement and strategic networking; foster the physician-scientist culture on an international scale; promote diversity; and importantly, build a community.

## Parting thoughts

Physician-scientists contribute to a critical niche in the international community, in striking display through the COVID-19 pandemic. Their intense curiosity coupled with knowledge of scientific principles, rigorous investigation, and a full understanding of the pathophysiology of disease has yielded rich advances in benefiting human health. Yet, this career path seems to us in peril around the globe. We believe that broad implementation of existing and novel strategies to support the training and career success of generations of physician-scientists might increase their recruitment, reduce attrition, and further fortify this exceptionally rewarding career path that is vital to the continued health and well-being of our societies.

We do not consider this article all-encompassing, and it is impossible to capture the many nuances in training physician-scientists at many different career stages. We hope that this piece will raise awareness of the common challenges and opportunities for physician-scientists at the international level and spark dialog, discussion, and collaboration in supporting this critical career sector.
